# Measurement properties of the USER-Participation Restriction subscale and PROMIS^®^ Ability to Participate in Social roles and Activities in in- and outpatient rehabilitation populations

**DOI:** 10.1007/s11136-026-04271-3

**Published:** 2026-06-05

**Authors:** B. M. P. Mourits, E. W. M. Scholten, J. A. de Graaf, R. J. E. M. Smeets, J. Nachtegaal, C. E. de Boer, I. J. W. van Nes, M. F. Reneman, D. M. Oosterveer, L. Valk-Kleibeuker, E. W. J. Agterhof, L. D. Roorda, J. M. A. Visser-Meily, M. W.M. Post

**Affiliations:** 1 Center of Excellence for Rehabilitation Medicine, UMC Utrecht Brain Center, University Medical Center Utrecht, and De Hoogstraat Rehabilitation, Utrecht, The Netherlands; 2 Department of Rehabilitation, Physical Therapy Science & Sports, UMC Utrecht Brain Center, University Medical Center Utrecht, Utrecht, The Netherlands; 3 Department of Rehabilitation Medicine, Care and Public Health Research Institute, Faculty of Health, Medicine & Life Sciences, Maastricht University, Maastricht, The Netherlands; 4 CIR Clinics In Revalidatie, Eindhoven, The Netherlands; 5 Pain in Motion International Research Group, Maastricht, The Netherlands; 6 Heliomare Rehabilitation Center, Research and Development, Wijk aan Zee, The Netherlands; 7 Merem Medical Rehabilitation, Hilversum, The Netherlands; 8 Department of Rehabilitation, Sint Maartenskliniek, Nijmegen, The Netherlands; 9 Department of Rehabilitation Medicine, Center for Rehabilitation, University Medical Center Groningen, University of Groningen, Groningen, The Netherlands; 10 Basalt, Leiden/The Hague, The Netherlands; 11 Department of Rehabilitation Medicine, Alrijne Hospital, Leiden, The Netherlands; 12 Department of Rehabilitation Medicine, Maasstad Hospital, Rotterdam, The Netherlands; 13 De Hoogstraat Rehabilitation, Utrecht, the Netherlands; 14 Amsterdam Rehabilitation Research Center, Reade, Amsterdam, The Netherlands

**Keywords:** Social participation, Patient-reported outcome measures, Rehabilitation, Reproducibility of results, Patient outcome assessment, Minimal clinically important difference

## Abstract

**Purpose:**

Participation is a key rehabilitation outcome. However, there is limited evidence on the measurement properties of patient-reported outcome measures (PROMs) that assess participation in rehabilitation settings. Therefore, this study evaluated the test-retest reliability and responsiveness of two widely used PROMs: the Utrecht Scale for Evaluation of Rehabilitation – Participation (USER-P) Restriction subscale and the Patient-Reported Outcomes Measurement Information System Ability to Participate in Social Roles and Activities 4-item short form (PROMIS-APS-SF) in inpatient and outpatient settings.

**Methods:**

In this multicentre prospective cohort study, inpatients and outpatients completed PROMs at the start of rehabilitation (T0), after six months (T1), and two weeks thereafter (T2). Test–retest reliability (T1-T2) was evaluated using intraclass correlation coefficients (ICCs), Bland-Altman plots, and the smallest detectable change (SDC). Responsiveness (T0-T1) was examined using effect sizes, area under the curve (AUC), and the minimal important change (MIC) based on the Global Rating of Change scale.

**Results:**

A total of 553 patients completed PROMs at T0–T1, of whom 168 also completed them at T2. Scores on both PROMs demonstrated sufficient test-retest reliability (ICC > 0.70) across both rehabilitation settings. Moderate to large effect sizes were found, except for the PROMIS-APS-SF scores in inpatients, which showed a small effect size. The USER-P Restriction scores achieved sufficient AUC values for inpatients (0.71) and outpatients (0.72). At group level, MIC values exceeded the SDC for both PROMs, but only few did at individual level.

**Conclusion:**

Scores on both PROMs appeared appropriate for evaluating participation outcomes at group level within rehabilitation settings, with the USER-P Restriction scores showing better responsiveness among inpatients. However, the use of these scores for evaluating individual participation goals seems limited.

**Supplementary Information:**

The online version contains supplementary material available at 10.1007/s11136-026-04271-3.

## Abstract (plain language summary)

Participation is often an important goal in rehabilitation for patients, but there is limited evidence how well different patient-reported outcome measures (PROMs) capture changes in participation during inpatient and outpatient treatment. This study used two commonly participation PROMs in rehabilitation: the USER-P Restriction subscale and the PROMIS-APS short form. Researchers examined whether the PROMs were consistent in stable participants (test-retest reliability), and whether they could detect meaningful changes in participation over time (responsiveness). In rehabilitation patients, scores on both PROMs showed sufficient reliability and adequate responsiveness across inpatient and outpatient settings. Changes in USER-P Restriction scores were larger among inpatients, likely reflecting changes in activity-based participation, while changes in PROMIS-APS-SF scores were lower, possibly capturing changes in broader social participation. At group level, meaningful change could be reliably detected, but at individual level, measurement error was too high to reliably detect these changes in individual patients. Overall, these findings suggest that both PROMs are suitable for evaluating participation outcomes in rehabilitation settings on group level and that the choice of PROM should match the specific aspect of participation that needs to be measured and evaluated.

**Supplementary Information:**

The online version contains supplementary material available at 10.1007/s11136-026-04271-3.

## Introduction

Improving participation is one of the main goals of multidisciplinary rehabilitation in healthcare, for people with disabilities due to various health conditions [1]. The International Classification of Functioning (ICF) defines participation as ‘involvement in a life situation’ [2]. Participation reflects active engagement in personally meaningful activities across domestic life, work, and community settings, capturing the broader impact of rehabilitation on an individual’s daily life and societal integration [3, 4]. Since participation is a core outcome of rehabilitation, evaluating it from the patient’s perspective is essential for determining the effectiveness of rehabilitation interventions.

In clinical practice and research, a wide range of generic Patient-Reported Outcome Measures (PROMs) is available to assess restrictions in participation and monitor change over time across diverse rehabilitation populations [5–8]. While many of these PROMs have been extensively studied for validity and reliability, much less is known about their responsiveness, especially in multidisciplinary rehabilitation treatment [9–11]. Two widely used participation PROMs are the Utrecht Scale for Evaluation of Rehabilitation-Participation (USER-P) Restriction subscale [12] and the Patient Reported Outcomes Measurement Information System Ability to Participate in Social Roles and Activities (PROMIS-APS) [13, 14]. The USER-P is often used in Dutch rehabilitation settings as a standard measure of participation, while the PROMIS short forms are increasingly being adopted in healthcare as part of efforts to harmonize outcome measurement and enable more precise patient-reported outcome monitoring [15]. Previous research has provided evidence supporting sufficient psychometric properties, including internal consistency and construct validity, of scores derived from both PROMs in various rehabilitation populations [16–21]. In our previous cross-sectional study, scores on the USER-P Restriction and PROMIS-APS 4-item short form (SF) showed similar internal consistency, construct validity, and discriminative validity in former rehabilitation patients [22]. However, evidence on responsiveness of both PROM scores during rehabilitation is lacking. Responsiveness refers to the ability of test scores to detect change over time in a specific context [23]. Such change can be quantified using distribution-based approaches as well as anchor-based approaches, which assess whether observed changes are meaningful from the patient’s perspective [24]. Establishing test-retest reliability is crucial to quantify measurement error of the scores and to determine whether meaningful changes are likely to exceed random variation in rehabilitation [25].

Because responsiveness may vary across care contexts, it is important to examine measurement properties of PROM scores within inpatient and outpatient rehabilitation settings [24, 26]. Inpatient rehabilitation primarily focuses on regaining independence and mobility in patients with acute onset conditions, whereas outpatient rehabilitation mainly focuses on social participation and coping with their condition in patients with more chronic conditions. Addressing these differences may inform the selection of a PROM for evaluating participation as a rehabilitation outcome in specific care contexts [27]. Therefore, the aim of this study was to estimate the test-retest reliability and responsiveness of scores on the USER-P Restriction and PROMIS-APS-SF in inpatient and outpatient rehabilitation populations, and to evaluate their measurement properties within each context.

## Methods

### Study design

As part of the Measurement of Outcomes of Rehabilitation in the Netherlands (MUREVAN) research project, this multicentre prospective cohort study was performed in 14 Dutch rehabilitation institutions. Participants completed a selection of PROMs, including the USER-P Restriction and the PROMIS-APS-SF, at three time points: at the start of rehabilitation treatment (T0), six months thereafter (T1) to assess responsiveness, and two weeks after T1 (T2) to assess test-retest reliability.

This study was not subject to the Dutch Medical Research Involving Human Subjects Act (WMO) and therefore did not require approval from an accredited Medical Ethics Committee (METC) in the Netherlands. To ensure compliance with legal and regulatory standards, including informed consent, data management, privacy, and ethical conduct, an independent quality check was carried out by the University Medical Centre Utrecht (UMCU) (date: 20-01-2023; reference number: 212217652). All participating institutions granted site-specific approval to conduct the study. We conducted the study in compliance with the Declaration of Helsinki [28].

### Setting and participants

Inpatients and outpatients with various diagnoses were recruited from the 14 Dutch rehabilitation institutions participating in the study. These included eight medical specialized rehabilitation centres (both in- and outpatient), three university hospitals (outpatient), two general hospitals (outpatient), and one outpatient rehabilitation centre. Inclusion criteria were: at least 18 years old, scheduled for a multidisciplinary treatment of minimal four weeks, and a diagnosis in one of the following rehabilitation groups: acquired brain injury (ABI; e.g., stroke, traumatic brain injury and brain tumor), spinal cord injury (SCI), chronic musculoskeletal pain disorder, neurological disorder (e.g., multiple sclerosis and neuromuscular diseases), or oncological conditions. Participants were excluded if they were unable to complete PROMs or had a rapidly progressive condition with a short life expectancy. Sample size estimates were based on quality criteria for validation studies, practical feasibility and potential dropouts [29]. To ensure sufficient statistical power for the responsiveness analyses, the baseline targets (T0) were 200 inpatients (100 each for ABI and SCI) and 500 outpatients (125 each for ABI, chronic musculoskeletal pain disorder, neurological disorder, and oncological conditions). For test-retest reliability at T2, the target was 200 participants: 50 inpatients and 150 outpatients. These participants were consecutively sampled based on completion of the follow-up assessment (T1).

### Procedures

Rehabilitation physicians of the participating institutions invited eligible patients either at the start of their rehabilitation treatment (inpatients) or during the intake before treatment (outpatients). Patients received an information letter and, if interested, their contact details were forwarded to the researcher. The researcher then contacted patients via email to confirm willingness to participate. According to the patients’ preference, the researcher sent a personalised link from the Castor Electronic Data Capture system (Castor EDC), or a paper version to their home address. Once the completed informed consent and questionnaire were received, the researcher sent a personalised Castor EDC link to the participant’s physician to collect disease-related characteristics. Follow-up questionnaires (T1 and T2) were sent in the same way as the baseline questionnaire. After each invitation, up to three reminders were sent via email or phone over a three-week period. Participant recruitment took place between March 2023 and October 2024. Final follow-up measurements were completed in April 2025.

### Outcome measures

#### USER-P

The USER-P assesses participation in three subscales: frequency, restriction and satisfaction [12]. In this study, we used the restriction subscale, which assesses the experienced restrictions in performing activities such as work, housekeeping, sport, and social interactions. The subscale consists of 11 items, each scored on a four-point scale ranging from ‘not possible’ to ‘without difficulty’. For example, one item asks: *‘Does your illness or condition currently limit your outdoor mobility*,* such as: driving a car*,* travelling by bus or train*,* cycling to work or going shopping?’*. The sum score of items is linearly transformed into a total score ranging from 0 to 100, with higher scores indicating a more favourable outcome (less restricted). Scores on the USER-P have demonstrated good validity and reliability in various rehabilitation populations [16, 30, 31].

#### PROMIS-APS-SF

The 4-item SF application of the PROMIS-APS v2.0 item bank assesses the perceived ability to perform usual social roles and activities by rating the limitation frequency [13]. Items are scored on a five-point scale ranging from ‘never’ to ‘always’. For example, one item asks: ‘*I have trouble doing all of my regular leisure activities with others’*. A T-score is calculated by transforming the sum score of items using a concordance table, ranging from 27.5 up to 64.2 [32]. Higher scores indicate more ability to participate. Scores on the PROMIS-APS item bank have demonstrated adequate internal consistency, good construct validity, and moderate to good responsiveness in various populations [33–36].

#### Global rating of change (GRC) scale

The GRC scale is a single-item measure that assesses a participant’s perceived change in an outcome at follow-up (T1). It can be used as a reference standard to evaluate the ability of PROM change scores to discriminate between improved and unimproved participants, and to calculate a minimal important change (MIC) value [26]. In this study, the GRC question was: ‘*To what extent have your participation restrictions changed since the start of your rehabilitation program?*’ Responses were scored on a seven-point scale, ranging from ‘much worse’ to ‘much improved’.

#### Demographic and disease-related characteristics

At baseline, participants’ demographic variables included self-reported sex, age, education level, living situation (alone or together), cognitive functioning (problems in memory, attention and planning), and physical functioning (problems in self-care, toileting and walking). At follow-up, participants were asked if they still received rehabilitation treatment (either the initial treatment or follow-up treatment). Disease-related variables, reported by the physician at baseline, included diagnosis, treatment setting (inpatient or outpatient), and comorbidity which affected treatment (yes or no). To evaluate stability between T1 and T2 (presumption to examine test-retest reliability), a stability question was administered at T2, asking participants to rate the extent to which their general health and daily functioning had changed over the preceding two weeks.

### Statistical analysis

All analyses were conducted separately for inpatient and outpatient groups using IBM SPSS Statistics for Windows (Version 27.0; Armonk, NY).

To describe the responsiveness sample (T0-T1) and the test-retest sample (T1-T2), we used descriptive statistics: categorical variables as frequencies and proportions, and continuous variables as means and standard deviations (SD) or medians and interquartile range (IQR). We calculated descriptive statistics of the USER-P Restriction scores and PROMIS-APS-SF scores at T0 and T1 in the responsiveness sample and at T1 and T2 in the test-retest sample, including mean (± SD), minimal and maximal value, floor effect (% patients with the worst score), and ceiling effects (% patients with the best score). A floor or ceiling effect was considered as ≥ 15% of the participants who scored the minimum or maximum score, respectively [37]. Internal consistency was assessed with Cronbach’s alpha, considering values ≥ 0.70 as sufficient [29].

#### Test-retest reliability

We assessed test-retest reliability of scores on both PROMs in participants reporting ‘no’ or ‘little change’ on the stability question at T2, using the intraclass correlation coefficient (ICC, two-way mixed model, absolute agreement) with 95% confidence interval (95% CI). In addition, we performed a more strict sensitivity analysis by evaluating test-retest reliability in the subgroup of participants who reported ‘no change’ on the stability question. Reliability was considered sufficient at ICC ≥ 0.70 [29]. We evaluated measurement error with paired t-tests and Bland-Altman plots, with limits of agreement (LoA) calculated as mean difference ± 1.96SD of the mean difference [38]. The standard error of measurement (SEM) was calculated from the variance components obtained from the mixed-effects ANOVA model. Specifically, the SEM was derived from the combined residual and occasion variance components, representing the total measurement error. A 95% CI for the SEM was estimated using the chi-square distribution of the error variance. The smallest detectable change (SDC) at individual level was calculated as 1.96 × √2 × SEM. The SDC at the group level was calculated as SDC individual / √N [39]. A 95% CI for the SDC at individual level was derived from the 95% CI of the SEM. The SDC represents the smallest change in score that can be interpreted as a real change above measurement error. In this study, the SDC is used as a benchmark to interpret the minimal important change (MIC).

#### Responsiveness

We assessed the responsiveness of scores on both PROMs using distribution-based and anchor-based methods in participants who completed both PROMs and the GRC scale. Distribution-based methods included effect size (ES=mean change/SD at baseline) and standardized response mean (SRM=mean change/SD of change), with 0.2, 0.5, and 0.8 indicating small, moderate, and large effects [40]. Paired t-tests were performed to compare T0 and T1 scores, with statistical significance set at *p* < 0.05.

Anchor-based methods included evaluating the relationship between PROM change scores and the GRC scale, we calculated Spearman’s correlation coefficients (≥ 0.50 considered adequate) and assessing area under the curves (AUC) from the receiver operating characteristic (ROC) curve (≥0.70 considered adequate) [41]. For the ROC analyses, the GRC scale was dichotomized into an improved group (‘moderately improved’ to ‘much improved’) and an unimproved group (‘much worse’ to ‘slightly improved’).

#### Minimal important change (MIC)

The MIC is defined as the smallest change score considered important by patients [26]. The MIC was determined using three anchor-based methods, which are commonly described in the literature: mean change method (MIC mean) [42], ROC curve method (MIC ROC) [43], and predictive modelling method (MIC predictive) [44]. MIC mean was calculated as the mean change in participants reporting ‘moderately improved’ on the GRC scale. Using the dichotomized GRC, we determined the MIC ROC as the optimal ROC cut-off (smallest misclassifications), and MIC predictive was calculated using logistic regression with 95% CI.

A correlation of > 0.30 between change scores and the GRC scale was required to calculate MIC values [45]. To be considered a reliably meaningful change in individual patients and patient groups, the MIC must exceed the SDC value at both the individual and group levels [26].

## Results

In total, 733 participants met the inclusion criteria and completed the baseline questionnaire. Of these, 569 (78%) completed the six-month follow-up questionnaire (*n* = 569). For the test-retest assessment, 243 participants were invited, with a response rate of 73% (*n* = 178). For the responsiveness analyses, 16 participants were excluded due to missing data on the USER-P Restriction, PROMIS-APS-SF and/or GRC scale. For the test-retest analyses, 10 participants were excluded due to substantial health changes between T1 and T2. For both the responsiveness and test-retest sample, Table 1 presents the baseline demographic and disease-related characteristics, and Table 2 the descriptive statistics for the scores on the USER-P Restriction and PROMIS-APS-SF at T0, T1 and T2. At T0, inpatients and outpatients showed different mean scores of the USER-P Restriction, whereas PROMIS-APS-SF scores were comparable. Scores on both PROMs showed a large variation for inpatients. Across all measurement points and in both samples, scores on the USER-P Restriction and PROMIS-APS-SF demonstrated high internal consistency (α > 0.86). In inpatients, ceiling effects were observed in the scores of the USER-P Restriction at T1 (18.2%) and T2 (19.1%), and a floor effect was observed in the PROMIS-APS-SF scores at T0 (21.9%).

**Table 1:** Baseline demographic and disease-related characteristics

	Responsiveness sample (*n* = 553)						Test-retest sample (*n* = 168)						
	Inpatient (*n* = 137)			Outpatient (*n* = 416)			Inpatient (*n* = 47)				Outpatient (*n* = 121)		
Demographic variables	n	n (%) or mean ± SD		n	n (%) or mean ± SD		n	n (%) or mean ± SD			n	n (%) or mean ± SD	
Sex (female)	137	49 (35.8)		416	284 (68.3)		47	23 (48.9)			121	82 (67.8)	
Age (years)	137	61 ± 13.8		416	54 ± 12.9		47	64 ± 11.3			121	55 ± 12.1	
Education (high)^a^	136	93 (68.4)		416	315 (75.8)		47	31 (65.9)			121	87 (71.9)	
Living situation (alone)	136	32 (23.5)		416	67 (16.1)		47	14 (29.8)			121	21 (17.4)	
Days between T0-T1	122	193 (19.0)^b^		396	190 (16.0)^b^		N/A				N/A		
Days between T1-T2	N/A			N/A			47	16 (11.0)^b^			121	16 (9.0)^b^	
Disease-related variables													
Diagnostic group *Acquired brain injury* *Chronic pain disorder* *Spinal cord injury* *Neurological disorder* *Oncology*	137	85 (62.0) N/A 52 (38.0) N/A N/A		416	125 (30.0) 100 (24.0) N/A 81 (19.5) 110 (26.4)		47	34 (72.3) N/A 13 (27.7) N/A N/A			121	29 (24.0) 34 (28.1) N/A 22 (18.2) 36 (29.8)	
Rehabilitation completed at T1 (yes)	135	82 (60.7)		386	293 (75.9)		46	29 (63.0)			101	76 (75.2)	
Cognitive functioning^c^ *Memory (no problems)* *Attention (no problems)* *Planning (no problems)*	136	80 (58.8) 78 (57.4) 83 (61.0)		416	131 (31.5) 95 (22.8) 112 (26.9)		46	31 (67.4) 29 (63.0) 29 (63.0)			121	41 (33.9) 27 (22.3) 35 (28.9)	
Physical functioning^c^ *Self-care (no problems)* *Toileting (no problems)* *Walking (no problems)*	136	39 (28.7) 56 (41.2) 24 (17.6)		416	261 (62.7) 346 (83.2) 139 (33.4)		46	11 (23.4) 17 (37.0) 6 (13.0)			121	78 (64.5) 102 (84.3) 38 (31.4)	
Comorbidity (yes)	137	55 (40.1)		416	85 (20.4)		47	15 (31.9)			121	26 (21.5)	

SD: standard deviation; N/A: not applicable; neurological disorder: mainly progressive diseases such as multiple sclerosis and neuromuscular diseases; ^a^ ≥ secondary education; ^b^ median (IQR); ^c^ self-reported outcomes; no problems: patients who experienced no problems in the specific domain.

**Table 2:** Descriptive statistics for scores on the USER-P Restriction and PROMIS-APS-SF at T0 and T1 (responsiveness sample) and at T1 and T2 (test-retest sample)

	Responsiveness sample (*n* = 553)			
	USER-P Restriction		PROMIS-APS-SF	
	Inpatient (*n* = 137)	Outpatient (*n* = 416)	Inpatient (*n* = 137)	Outpatient (*n* = 416)
*T0*				
Mean ± SD	45.8 ± 22.7	67.0 ± 16.9	40.5 ± 10.7	40.2 ± 5.9
Min-max value	0-100	12–100	27.5–64.2	27.9–64.2
Floor (% min. scores)	0.7	0.0	21.9	5.5
Ceiling (% max. scores)	3.6	2.2	8.0	0.2
Cronbach’s alpha	0.90	0.86	0.92	0.88
*T1*				
Mean ± SD	70.5 ± 23.1	76.1 ± 17.2	44.8 ± 9.5	44.0 ± 7.1
Min-max value	18–100	18–100	27.5–64.2	27.5–64.2
Floor (% min. scores)	0.0	0.0	5.1	1.7
Ceiling % max. scores	18.2	10.1	8.0	3.1
Cronbach’s alpha	0.93	0.89	0.94	0.91

	Test-retest sample (*n *= 168)			
	USER-P Restriction		PROMIS-APS-SF	
	Inpatient (*n* = 47)	Outpatient (*n* = 121)	Inpatient (*n* = 47)	Outpatient (*n* = 121)
*T1*				
Mean ± SD	72.1 ± 22.3	75.3 ± 17	46.3 ± 9.4	43.8 ± 7.0
Min-max value	18.2–100	18.2–100	31.8–64.2	27.5–64.2
Floor (% min. scores)	0.0	0.0	0.0	1.7
Ceiling (% max. scores)	21.3	8.3	10.6	2.5
Cronbach’s alpha	0.89	0.89	0.96	0.90
*T2*				
Mean ± SD	73.5 ± 21.3	75.9 ± 17.1	46.7 ± 9.6	43.7 ± 7.0
Min-max value	26.7–100	20–100	27.5–64.2	27.5–64.2
Floor (% min. scores)	0.0	0.0	4.3	4.1
Ceiling (% max. scores)	19.1	7.4	6.4	2.5
Cronbach’s alpha	0.87	0.88	0.95	0.91

SD: standard deviation; USER-P: Utrecht Scale for Evaluation of Rehabilitation-Participation (range 0-100, higher score is more ability to participate); PROMIS-APS-SF: Patient Reported Outcomes Measurement Information System Ability to Participate in Social Roles and Activities Short form (range 27.5–64.2, higher score is more ability to participate); min: minimal; max: maximal.

### Test-retest reliability

**Fig. 1:**
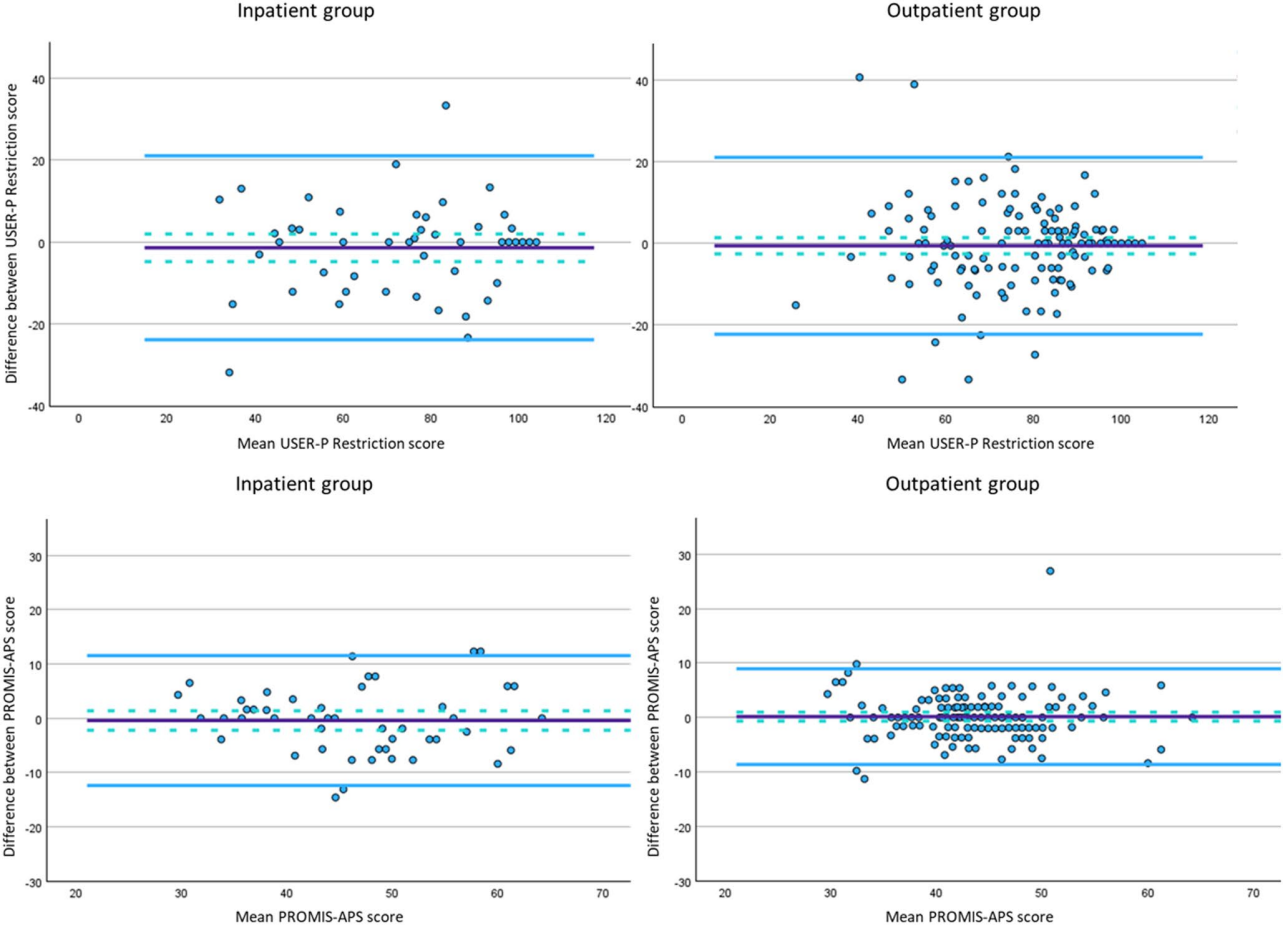
Bland-Altman plots for the inpatient group (left) and outpatient group (right) of the USER-P Restriction and PROMIS-APS-SF. Purple solid line depicts the mean difference score and light blue solid lines the limits of agreement. Light blue dashed lines are the 95% CI of the mean difference

Scores on both PROMs showed sufficient test-retest reliability in both inpatients and outpatients (ICC > 0.70) (Table 3). Variance components, SEM (95% CI) and corresponding SDC values at both individual (95%CI) and group level are presented in Table 3. No significant systematic difference (*p* > 0.05) was observed between T1 and T2 scores for either measure. Bland-Altman plots (Fig. 1) indicated wide but comparable LoA values (Table 3) across groups for both PROMs. Additional sensitivity analyses of the test-retest variables showed similar results, although the 95% CI’s were wider due to the smaller subgroup sample sizes (Online resource 1).

**Table 3:** Test-retest reliability, paired t-test, and SDC of scores on the USER-P Restriction and PROMIS-APS-SF (T1 versus T2)

	Test-retest sample (*n* = 168)			
	USER-P Restriction		PROMIS-APS-SF	
	Inpatient (*n* = 47)	Outpatient (*n* = 121)	Inpatient (*n* = 47)	Outpatient (*n* = 121)
Variance components				
*Patient*	409.126	229.770	71.658	38.915
*Occasion*	15.765	20.291	6.487	3.514
*Residual*	49.807	40.090	12.151	6.542
ICC (95% CI)	0.86 (0.77–0.92)	0.79 (0.71–0.85)	0.80 (0.66–0.88)	0.80 (0.72–0.85)
SEM (95% CI)	8.1 (6.8–10.3)	7.8 (7.0-8.8)	4.3 (3.6–5.5)	3.2 (2.8–3.6)
Mean diff score ± SD	−1.4 ± 11.5	−0.61 ± 11.0	−0.43 ± 6.1	0.16 ± 4.5
95%CI diff (*p*-value)	−4.8–2.0 (0.41)	−2.6–1.4 (0.54)	−2.2–1.4 (0.63)	−0.65–0.96 (0.70)
LoA	−23.8–21.1	−22.3–21.0	−12.4–11.5	−8.6–8.9
SDC individual (95% CI)	22.5 (18.9–28.6)	21.5 (19.3–24.4)	12.0 (10.1–15.2)	8.8 (7.9–9.9)
SDC group	3.3	2.0	1.8	0.8

USER-P: Utrecht Scale for Evaluation of Rehabilitation-Participation (range 0-100, higher score is more ability to participate); PROMIS-APS-SF: Patient Reported Outcomes Measurement Information System Ability to Participate in Social Roles and Activities Short form (range 27.5–64.2, higher score is more ability to participate); ICC: intra-class correlation coefficient; CI: confidence interval; SEM: standard error of measurement; diff: difference; SD: standard deviation; LoA: limits of agreement; SDC: smallest detectable change.

### Responsiveness

**Fig. 2:**
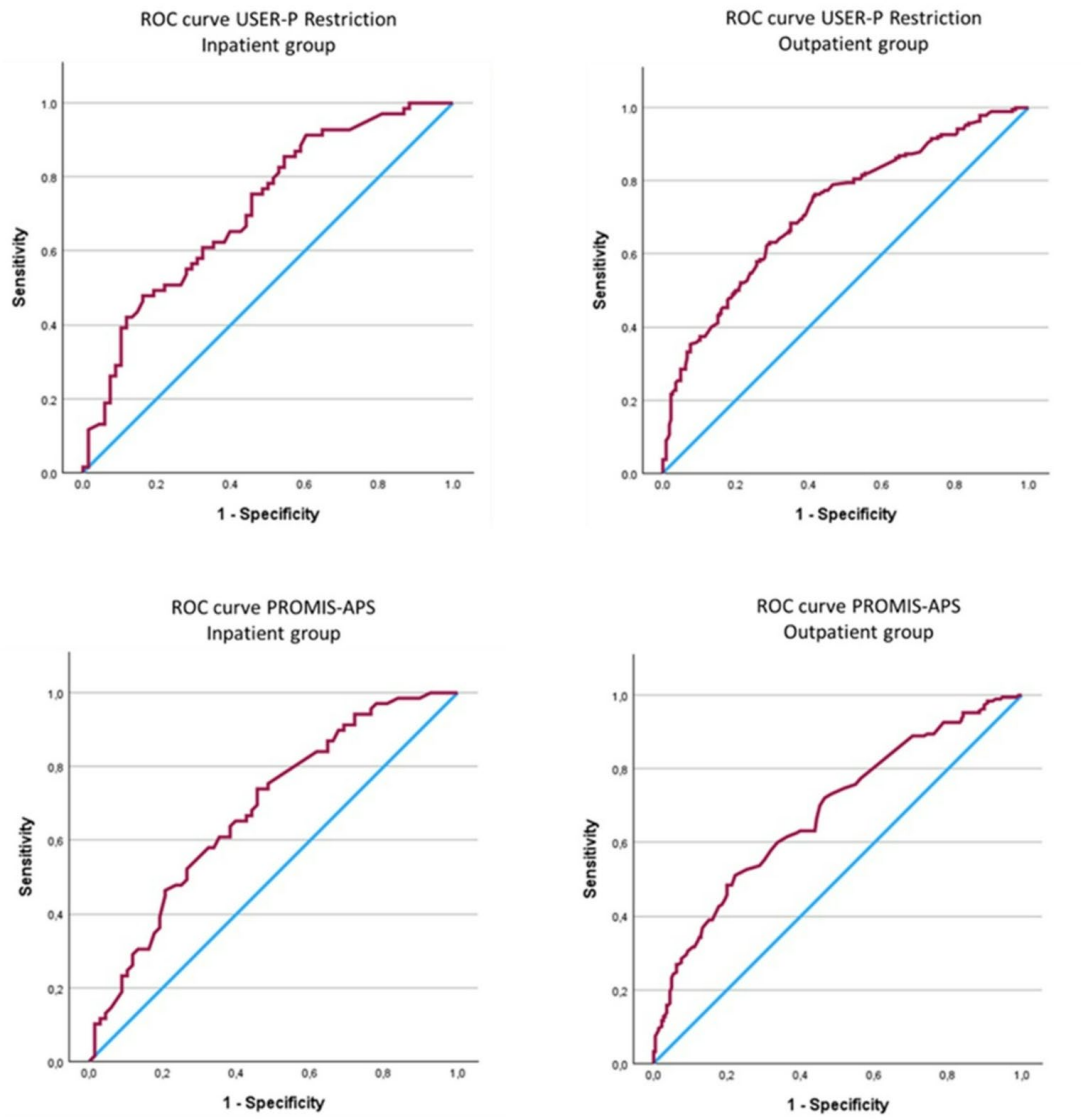
ROC curves of the USER-P Restriction and PROMIS-APS-SF change scores (T0 and T1) of the inpatient group (left) and outpatient group (right). Blue lines depicts the reference line and the red lines the change score

Table 4 presents the ES, SRM, AUC, and correlation with the GRC scale for scores on both PROMs in the responsiveness sample across rehabilitation settings. Mean changes between T0 and T1 scores were statistically significant across groups for both PROMs (*p* < 0.001). Scores on the USER-P Restriction showed large ES and SRM values in inpatients (1.0–1.1) and moderate in outpatients (0.54–0.55). Scores on the PROMIS-APS-SF showed small to moderate effects in inpatients (0.38–0.40) and moderate in outpatients (0.55–0.65). Scores on the USER-P Restriction showed sufficient discriminative ability, with AUC values of 0.71 for inpatients and 0.72 for outpatients, whereas scores on the PROMIS-APS-SF showed AUC values slightly below 0.70 (AUC = 0.68 for both inpatients and outpatients). Corresponding ROC curves are shown in Fig. 2. The correlations between both PROM change scores and the GRC scale were small, ranging from 0.30 for the PROMS-APS in inpatients to 0.42 for the USER-P Restriction in outpatients. Therefore, none of the correlations were considered adequate (≥0.50) in these settings.

**Table 4:** Responsiveness outcomes of scores on the USER-P Restriction and PROMIS-APS-SF: effect size, standardized response mean, area under the curve, and correlation with the global rating of change scale

	Responsiveness sample (*n* = 553)			
	USER-P Restriction		PROMIS-APS-SF	
	Inpatient (*n* = 137)	Outpatient (*n* = 416)	Inpatient (*n* = 137)	Outpatient (*n* = 416)
Mean change ± SD	24.7 ± 24.0**	9.1 ± 16.5**	4.3 ± 11.1**	3.8 ± 7.0**
Effect size	1.10	0.54	0.40	0.65
SRM	1.00	0.55	0.38	0.55
AUC (95% CI)	0.71 (0.63–0.80)	0.72 (0.67–0.77)	0.68 (0.59–0.77)	0.68 (0.63–0.74)
Correlation GRC	0.35	0.42	0.30	0.36

USER-P: Utrecht Scale for Evaluation of Rehabilitation-Participation (range 0-100, higher score is more ability to participate); PROMIS-APS-SF: Patient Reported Outcomes Measurement Information System Ability to Participate in Social Roles and Activities Short form (range 27.5–64.2, higher score is more ability to participate); SD: standard deviation; SRM: standardized response mean; AUC: area under the curve; GRC: global rating of change; ** p value < 0.001

### Minimal important change

Approximately 50% of both inpatients and outpatients were classified as ‘unimproved’ according to the dichotomized GRC scale. In inpatients, MIC values ranged from 24.0 to 34.9 for scores on the USER-P Restriction and from 1.8 to 7.2 for scores on the PROMIS-APS-SF (Table 5). In outpatients, MIC values ranged from 5.5 to 15.0 for scores on the USER-P Restriction and from 4.4 to 7.0 for scores on the PROMIS-APS-SF. None of the MIC values exceeded the SDC at individual level, except for the MIC values for scores on the USER-P Restriction in inpatients. For both groups, all MIC values exceeded the SDC value at group level for scores on both PROMs.

**Table 5:** USER-P Restriction and PROMIS-APS-SF change scores (T0 vs. T1) for each response option of the global rating of change scale and the minimal important change values per method

	Responsiveness sample (*n* = 553)								
	USER-P Restriction change scores				PROMIS-APS-SF change scores				
GRC response options	Inpatient (*n* = 137)		Outpatient (*n* = 416)		Inpatient (*n* = 137)		Outpatient (*n* = 416)		
	n (%)	Mean ± SD	n (%)	Mean ± SD	n (%)	Mean ± SD	n (%)		Mean ± SD
*Much worse*	3 (2.2)	1.4 ± 4.4	3 (0.7)	2.9 ± 18.4	3 (2.2)	0.6 ± 1.0	3 (0.7)		2.2 ± 7.9
*Moderately worse*	14 (10.2)	5.2 ± 15.4	14 (3.4)	−4.8 ± 17.5	14 (10.2)	1.1 ± 8.4	14 (3.4)		−1.4 ± 6.6
*Slightly worse*	10 (7.3)	16.8 ± 25.7	23 (5.5)	8.5 ± 15.7	10 (7.3)	−3.2 ± 14.4	23 (5.5)		0.2 ± 5.9
*No change*	16 (11.7)	28 ± 28.4	71 (17.1)	−0.5 ± 10.8	16 (11.7)	3.2 ± 14.6	71 (17.1)		1.8 ± 6.2
*Slightly improved*	25 (18.2)	15.4 ± 17.1	115 (27.6)	5.5 ± 13.6	25 (18.2)	0.1 ± 10.2	115 (27.6)		2.4 ± 5.4
Moderately improved	32 (23.4)	34.9 ± 22.7	112 (26.9)	12.4 ± 15.3	32 (23.4)	7.2 ± 10.1	112 (26.9)		4.4 ± 5.8
Much improved	37 (27.0)	32.1 ± 23.0	78 (18.8)	21.5 ± 17.2	37 (27.0)	8.5 ± 9.2	78 (18.8)		9.1 ± 8.5
MIC values									
*MIC mean ± SD*	34.9 ± 22.7		12.4 ± 15.3		7.2 ± 10.1		4.4 ± 5.8		
*MIC ROC (sens; spec)*	33.1 (0.48;0.84)		5.5 (0.76;0.58)		1.8 (0.74;0.54)		5.2 (0.51;0.78)		
MIC mean ± SD	24.0 (12.8–35.8)		15.0 (11.5–19.4)		4.4 (-1.9–10.5)		7.0 (5.1–9.7)		

USER-P: Utrecht Scale for Evaluation of Rehabilitation-Participation (range 0–100, higher score is more ability to participate); PROMIS-APS-SF: Patient Reported Outcomes Measurement Information System Ability to Participate in Social Roles and Activities Short form (range 27.5–64.2, higher score is more ability to participate); GRC: global rating of change; SD: standard deviation; MIC: minimal important change; ROC: receiver operating characteristic; sens: sensitivity; spec: specificity; 95% CI 95% confidence interval.

## Discussion

In this study, we estimated the test-retest reliability and responsiveness of scores on the USER-P Restriction and the PROMIS-APS-SF to evaluate their measurement properties in inpatient and outpatient rehabilitation populations. Scores on both PROMs demonstrated sufficient test-retest reliability in both inpatient and outpatient groups, indicating consistent measurement, however measurement error was relatively high at individual level. In outpatients, both PROM change scores had moderate effect sizes, however in inpatients the change scores on the USER-P Restriction showed a large effect size and for change scores on the PROMIS-APS-SF a small effect size. For both groups, change scores on the USER-P Restriction showed a slightly better ability to discriminate between improved and unimproved patients than for scores on the PROMIS-APS-SF. At group level, MIC values for scores on both PROMs exceeded the SDC in both inpatient and outpatient groups, suggesting that meaningful change can be detected beyond measurement error at this level. Across different MIC methods, values varied considerably for both PROMs in both groups, with the highest consistency found for the PROMIS-APS-SF scores in outpatients.

In our study, the test-retest reliability of scores on both PROMs was sufficient (ICC = 0.79–0.86) for both the inpatient and outpatient group. This aligns with previous research, in which scores on the USER-P Restriction demonstrated an ICC of 0.85 in outpatients undergoing rehabilitation, and scores on the PROMIS-APS showed ICC’s ranging from 0.75 to 0.87 across various chronic disease populations [12, 46–48]. However, these PROMIS-APS values were obtained using a computer adaptive test (CAT) of the PROMIS-APS item bank rather than a short form, meaning that the number and selection of items varied per respondent. Nonetheless, other research indicated that ICC values for CATs are generally similar to those of the corresponding short forms [49]. Overall, our findings further support the evidence that scores on both PROMs are reliable in rehabilitation settings.

In both the inpatient and outpatient group, the relatively high magnitude of the SEM and SDC for scores on both PROMs indicates that, at the individual level, detecting true change is challenging when using the conventional 95% confidence interval. Using a less strict threshold, as suggested by Peipert et al. (2022), with a 68% confidence interval (likely change index), lowers the SDC (USER-P Restriction: 11.5 and 11.0; PROMIS-APS: 6.1 and 4.5, for inpatients and outpatients respectively) and aligns more closely with the mean observed changes and MIC values in our study [50]. In line with our responsiveness findings, the 68% confidence interval may therefore provide a more clinically informative threshold for individual interpretation, particularly when detecting meaningful change is prioritized. However, this approach entails accepting a higher probability that some observed changes reflect measurement variability rather than true clinical improvement, underscoring the trade-off between statistical certainty and clinical sensitivity. With respect to distribution-based responsiveness in outpatients, scores on both PROMs showed moderate effect sizes (ES = 0.54; SRM = 0.55 and ES = 0.66; SRM = 0.54, respectively), which were somewhat higher than previously reported [30, 51–54]. Previous studies found small to moderate effect sizes for scores on the USER-P Restriction. A lower value (ES = 0.27) was observed in a study including a higher proportion of patients with neuromuscular diseases, who typically show less physical improvement, whereas another study including a more heterogeneous diagnostic mix (similar to our sample) reported a similar effect size (ES = 0.49) [30, 51]. Previously reported effect sizes for scores on the PROMIS-APS (ES = 0.16–0.39) were lower than in our study, likely reflecting study population differences and follow-up duration, as two of the three studies focused on patients with chronic pain and had a four-week follow-up [52–54]. When interpreting the PROMIS-APS-SF change scores in our study in light of the Dutch reference values, it becomes evident that most patients shift from levels corresponding to moderate/mild limitations toward mild or no limitations [55]. These findings, together with our clinical experience, suggest that participation levels may continue to improve over longer treatment periods, and that scores on both PROMs may be more responsive when assessed over extended intervals [56]. At the same time, a portion of patients already showed high baseline participation scores, naturally limiting the room for detectable improvement in some subgroups. Moreover, rehabilitation may also have a limited effect on participation, given the influence of external factors beyond the control of healthcare professionals, such as living alone, or the level of support provided by an employer [57].

With regard to distribution-based responsiveness in inpatients, scores on the USER-P Restriction showed the largest effect size (ES = 1.1; SRM = 1.0), with low baseline scores compared to outpatients, and ceiling effects at T1. Although ceiling effects have been reported previously, these were mainly observed in former outpatient rehabilitation patients [16, 31, 58]. The relatively large responsiveness in inpatients is therefore unexpected, particularly because the USER-P was developed for outpatient rehabilitation, where participation is a more explicit treatment goal [12, 17]. The large improvements could be explained by the continued rehabilitation interventions after discharge to home, as approximately 40% were still receiving rehabilitation treatment at follow-up. In contrast, scores on the PROMIS-APS-SF showed neither a comparable effect size nor ceiling effects. Despite a floor effect at T0, baseline scores were comparable to those of outpatients. These differences could be explained by dissimilarities in item content and response options [22]. USER-P Restriction items are concrete and activity-based, with functional limitation-based response categories, as the PROMIS-APS-SF items address broader perceptions of participation with frequency-based responses. Additional analyses of item response distributions (Online resource 2) support this proposed explanation. In inpatients and outpatients, scores on the USER-P Restriction show greater variability between relatively easy and more demanding activities, whereas scores on the PROMIS-APS-SF show more uniform patterns across items. Additionally, the USER-P Restriction includes a ‘not applicable’ response, allowing differentiation between participation restrictions that are caused by their condition and those that are not, potentially increasing response variability. Moreover, achieving the highest response category seems easier in the USER-P Restriction than selecting'never' in PROMIS-APS-SF. Overall, scores on the USER-P Restriction may be more responsive to change in activity-based participation, while scores on the PROMIS-APS-SF capture a broader and more stable perceptions of social participation [22]. However, recent expansions of the PROMIS-APS item bank, including improved coverage at lower participation levels and the addition of a ‘Skip’ option, may enhance its suitability for inpatient rehabilitation in future applications [59, 60].

With regard to anchor-based responsiveness for both inpatient and outpatient groups, scores on the USER-P Restriction just barely showed a sufficient AUC value, whereas scores on the PROMIS-APS-SF were close to sufficient. Moreover, scores on neither PROM revealed a strong correlation with the GRC scale in both groups. These results are consistent with a previous study that evaluated the responsiveness and MIC of scores on the PROMIS-APS CAT in individuals with kidney disease [61]. These findings suggest that scores on the PROMs may be limited in their ability to discriminate between patients who reported improvements in participation over time and those who did not in these populations. However, it could be noted that the GRC scale may not serve as an optimal reference standard or that the GRC scale was not ideally formulated [62–65]. The GRC scale is susceptible to response and recall biases, and as a single measure, it may lack the reliability and validity required to capture a complex, multifaceted construct such as participation [5, 66, 67].

In our study, MIC values exceeded the SDC at group level, however rarely at individual level in scores of both PROMs across groups. This indicates that scores of both PROMs have limited ability to reliably detect clinically meaningful change in single patients in inpatients and outpatients in rehabilitation, as observed score changes may be attributable to measurement error rather than true change [26, 68]. Only scores of the USER-P Restriction in inpatients exceeded the SDC at individual level, but the magnitude of these MIC values raises doubts about whether they truly represent a *minimal* important change. These findings align with previous research showing that scores of the USER-P and PROMIS-APS typically demonstrate MIC values below individual SDC threshold in various chronic disease populations [12, 30, 47, 48, 69]. Notably, the MIC values for scores on the PROMIS-APS-SF in our study were lower than those reported in a systematic review, suggesting that even small change scores may be meaningful to patients [25]. Overall, these results suggest that the MIC of scores on both PROMs are more suitable for group-level evaluation in research and quality assessments contexts than in daily practice with individual patients in rehabilitation.

The three different anchor-based methods revealed substantial variation in MIC values and large variability surrounding the MIC values in inpatients and outpatients. This variability may reflect methodological limitations as well as sample-related characteristics, including sample size, heterogeneity, and baseline dependency of MIC values [26, 70]. These findings raise concerns about the a single standardized MIC value for clinical and research application and underline persistent conceptual and methodological challenges in MIC estimation [71]. Further refinement in MIC methodology and potentially improvements in PROM measurement precision is required to obtain more reliable estimates that truly reflect *important* change from the patient’s perspective [72, 73]. In this context, more advanced approaches such as longitudinal CFA-based methods or adjusted predictive modelling may provide more precise estimates of MIC. However, in our study the distribution of participants classified as “improved” versus “not improved” was relatively balanced, reducing the potential benefit of using adjusted predictive modelling. Therefore, a simpler predictive modelling approach was applied to ensure feasibility and clinical interpretability. Consequently, the MIC values in our study should serve as indicative thresholds to support shared decision-making and facilitate discussions with patients about meaningful change in participation.

### Strengths and limitations

This is the first study to estimate the test-retest reliability and responsiveness of scores on the USER-P Restriction and PROMIS-APS-SF in a rehabilitation context, providing novel evidence to aid in the selection of PROMs for evaluating participation in different rehabilitation settings. A strength of this study is its multicentre prospective design, which included participants across treatment settings and institutions in the Netherlands. This enhances the external validity of the findings and supports their applicability to a broad rehabilitation population.

There are several limitations to address. First, our responsiveness evaluation was based solely on distribution-based analyses and on the reference standard GRC scale for the anchor-based analyses. This limited the extent to which comprehensive hypothesis testing could be performed and precluded a formal evaluation of longitudinal validity in this study [74]. However, previous research showed that the PROMIS-APS CAT showed sufficient longitudinal validity in patients with knee arthroplasty [75]. Moreover, it is important to highlight that all methods used to quantify responsiveness are influenced by contextual factors such as sample composition, assessment interval, and clinical characteristics. Consequently, the observed responsiveness results apply to the specific clinical and study context rather than as absolute properties of the measures. Secondly, MIC values were calculated based on single anchor-based methods for improvement only, as rehabilitation populations primarily show recovery rather than deterioration. Moreover, the number of patients reporting worsening in our study was too small for a reliable estimate of MIC deterioration. Finally, it should be noted that our MIC values were derived using a GRC scale, which assessed the amount of change rather than its perceived importance. Consequently, these MIC values do not represent a direct measure of minimal important change. For practical purposes, we used the response option 'moderately improved' as the cut-off point to define the minimal important change [76, 77]. However, there remains debate about whether this truly reflects the level of change that is important to patients, especially given the substantial variability in what different patients consider important and the potential influence of baseline scores [26, 78].

## Conclusion

Scores on both PROMs showed sufficient reliability in inpatient and outpatient rehabilitation populations. Responsiveness was generally adequate for both PROMs across settings, with a large effect size for change scores on the USER-P Restriction in inpatients and slightly better discrimination between improved and unimproved patients in both rehabilitation settings. Although measurement error was relatively high at the individual level, meaningful change could be detected reliably at group level in both PROMs for inpatients and outpatients. Given the ongoing methodological challenges in determining robust MIC values, caution is warranted when interpreting these values.

Overall, our findings suggest that scores of both PROMs are suitable for evaluating participation outcomes at group level in both rehabilitation settings. Change scores on the USER-P Restriction appear to reflect changes in activity-based participation, while change scores on the PROMIS-APS-SF seem to reflect changes in global social participation. Therefore, the choice of PROM should align with the specific aspects of participation that are intended to be measured and evaluated in the specific context.

## Supplementary Information

Below is the link to the electronic supplementary material.

Supplementary Material 1

Supplementary Material 2

## Data Availability

The dataset used and analysed during the current study is available from the corresponding author on reasonable request via the public data repository DataverseNL (10.34894/F5MIGX).
