# Supplementary material for: Measurement properties of the USER-Participation Restriction subscale and PROMIS® Ability to Participate in Social roles and Activities in in- and outpatient rehabilitation populations
Source: Qual Life Res. 2026 Jun 5;35(7):164. doi: 10.1007/s11136-026-04271-3 (PMC13241418; doi:10.1007/s11136-026-04271-3)
Supplement: Supplementary file 1 — Supplementary Material 1 [file 11136_2026_4271_MOESM1_ESM.pdf]

**Online resource 1.**

Table 1. Sensitivity analyses of the test-retest reliability, paired t-test, and SDC of scores on the USER-P Restriction and PROMIS-APS-SF (T1 versus T2) on a subgroup of the test-retest sample (participants with only 'no change' on the stability question)

| | **Test-retest sample (n=111)** | | | |
|---|---|---|---|---|
| | USER-P Restriction | | PROMIS-APS-SF | |
| | Inpatient (n=26) | Outpatient (n=85) | Inpatient (n=26) | Outpatient (n=85) |
| Variance components<br>*Patient*<br>*Occasion*<br>*Residual* | <br>392.174<br>16.167<br>46.738 | <br>208.212<br>17.946<br>28.441 | <br>71.491<br>7.293<br>15.704 | <br>42.393<br>3.907<br>7.777 |
| ICC (95%CI) | 0.86 (0.72-0.94) | 0.82 (0.74-0.88) | 0.76 (0.54-0.89) | 0.78 (0.69-0.85) |
| SEM (95%CI) | 7.9 (6.2-11.3) | 6.8 (6.0-8.0) | 4.8 (3.8-6.8) | 3.4 (3.0-4.0) |
| Mean diff score±SD | -1.4 (11) | 0.07 (9.6) | 0.17 (6.8) | 0.61 (4.8) |
| 95%CI diff<br>(p-value) | -6 – 3.1<br>(0.52) | -2.0 – 2.2<br>(0.94) | -2.5 – 2.9<br>(0.90) | -0.4 – 1.7<br>(0.25) |
| LoA | -23.4 – 20.6 | -18.8 – 18.9 | -13.1 – 13.5 | -8.9 – 10.1 |
| SDC individual (95%CI) | 22.0 (17.2-31.2) | 18.9 (16.6-22.0) | 13.3 (10.4-18.9) | 9.5 (8.3-11.1) |
| SDC group | 4.3 | 2.0 | 2.6 | 1.0 |

USER-P: Utrecht Scale for Evaluation of Rehabilitation-Participation (range 0-100, higher score is more ability to participate); PROMIS-APS-SF: Patient Reported Outcomes Measurement Information System Ability to Participate in Social Roles and Activities Short form (range 27.5-64.2, higher score is more ability to participate); ICC: intra-class correlation coefficient; CI: confidence interval; diff: difference; SD: standard deviation; LoA: limits of agreement; SDC: smallest detectable change.

**Article name:**

Comparison of measurement properties between USER-Participation Restriction subscale and PROMIS® Ability to Participate in Social Roles and Activities in in- and outpatient rehabilitation populations

**Journal name:**

Quality of Life Research

**Author names and affiliations:**

B.M.P. Mourits, MSc[1]; E.W.M. Scholten, PhD[1,2]; J.A. de Graaf, (MD) PhD[1,2]; R.J.E.M. Smeets, (MD) PhD[3,4,5]; J. Nachtegaal, PhD[6]; C.E. de Boer, (MD)[7]; I.J.W. van Nes, (MD) PhD[8]; M. F. Reneman, (MD) PhD[9]; D.M. Oosterveer, (MD) PhD[10,11]; L. Valk-Kleibeuker, (MD)[12]; E.W.J. Agterhof, (MD)[13]; L.D. Roorda, (MD, PT) PhD[14]; J.M.A. Visser-Meily, (MD) PhD[1,2]; M.W.M. Post, PhD[1]

[1] *Center of Excellence for Rehabilitation Medicine, UMC Utrecht Brain Center, University Medical Center Utrecht, and De Hoogstraat Rehabilitation, Utrecht, the Netherlands;*
[2] *Department of Rehabilitation, Physical Therapy Science & Sports, UMC Utrecht Brain Center, University Medical Center Utrecht, the Netherlands;*
[3] *Department of Rehabilitation Medicine, Care and Public Health Research Institute, Faculty of Health, Medicine & Life Sciences, Maastricht University, Maastricht, Netherlands;*
[4] *CIR Clinics In Revalidatie, Eindhoven, Netherlands;*
[5] *Pain in Motion International Research Group, Maastricht, Netherlands;*
[6] *Heliomare Rehabilitation Center, Research and Development, Wijk aan Zee, Netherlands*
[7] *Merem Medical Rehabilitation, Hilversum, The Netherlands;*
[8] *Department of Rehabilitation, Sint Maartenskliniek, Nijmegen, Netherlands;*
[9] *Department of Rehabilitation Medicine, Center for Rehabilitation, University Medical Center Groningen, University of Groningen, Groningen, Netherlands;*
[10] *Basalt, Leiden/The Hague, The Netherlands;*
[11] *Department of Rehabilitation Medicine, Alrijne Hospital, Leiden, The Netherlands;*
[12] *Department of Rehabilitation Medicine, Maasstad Hospital, Rotterdam, The Netherlands;*
[13] *De Hoogstraat Rehabilitation, Utrecht, the Netherlands;*
[14] *Amsterdam Rehabilitation Research Center, Reade, Amsterdam, The Netherlands.*

**Corresponding author:**

E.W.M. Scholten

Center of Excellence for Rehabilitation Medicine, UMC Utrecht Brain Center, University Medical Center Utrecht, and De Hoogstraat Rehabilitation, Utrecht, the Netherlands

Address: UMC Utrecht Brain Center, Heidelberglaan 100, 3584 CX, Utrecht, The Netherlands

Email: E.Scholten-2@umcutrecht.nl
