# Supplementary material for: Measurement properties of the USER-Participation Restriction subscale and PROMIS® Ability to Participate in Social roles and Activities in in- and outpatient rehabilitation populations
Source: Qual Life Res. 2026 Jun 5;35(7):164. doi: 10.1007/s11136-026-04271-3 (PMC13241418; doi:10.1007/s11136-026-04271-3)
Supplement: Supplementary file 2 — Supplementary Material 2 [file 11136_2026_4271_MOESM2_ESM.pdf]

Online resource 2:

Item response distributions USER-P Restriction and PROMIS-APS-SF

Fig 1. USER-P Restriction at T0 inpatient group

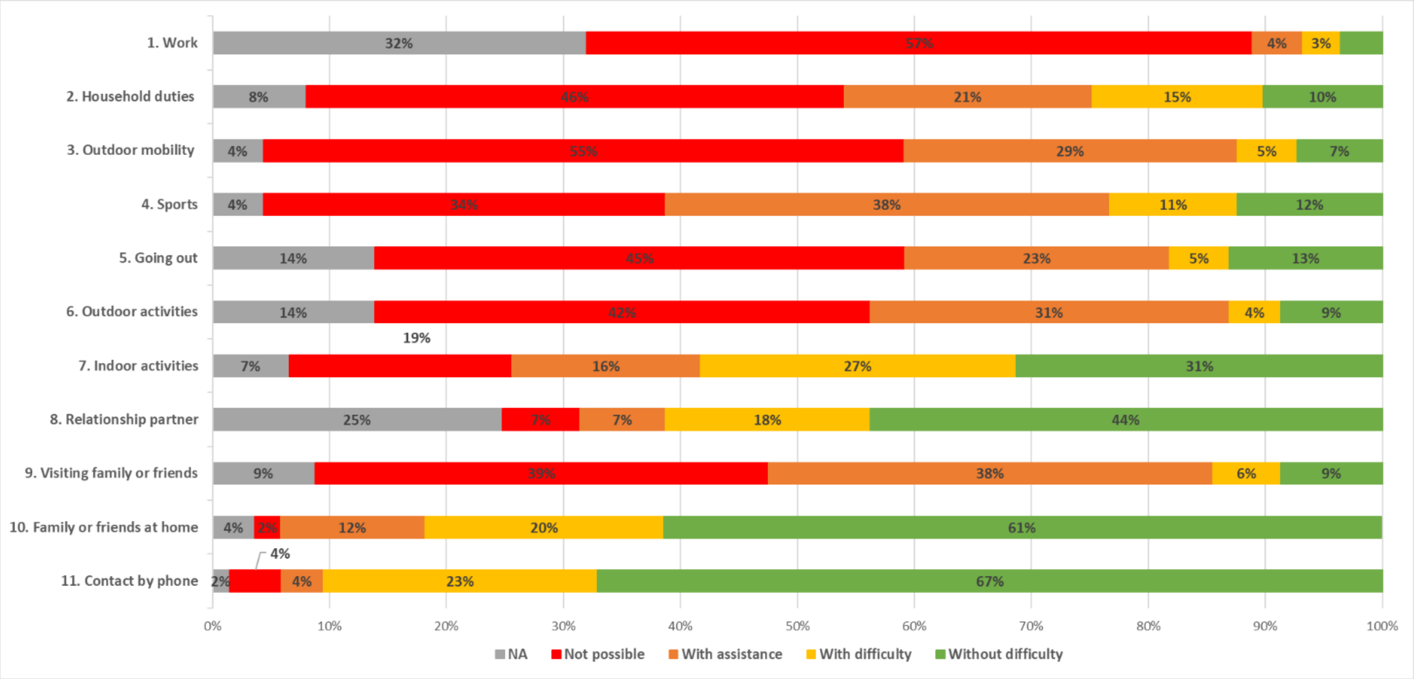

Fig 2. USER-P Restriction at T1 inpatient group

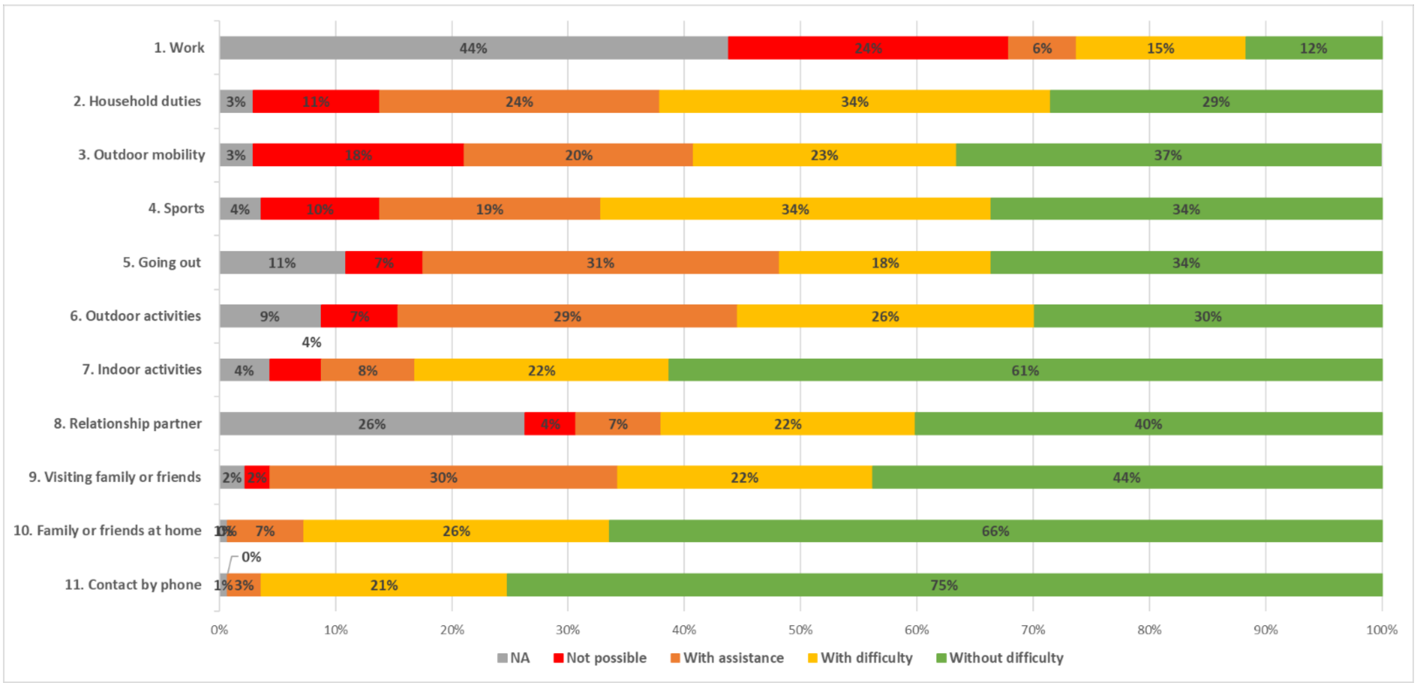

Fig 3. USER-P Restriction at T0 outpatient group

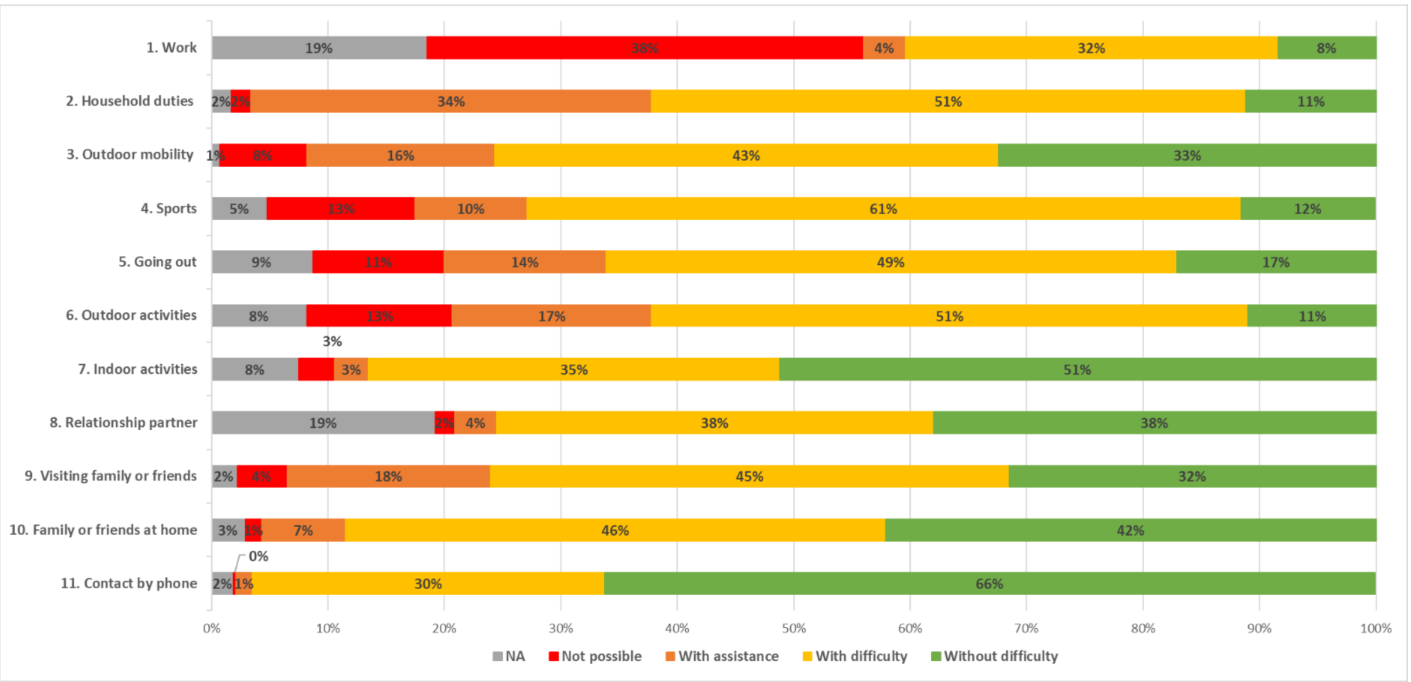

Fig 4. USER-P Restriction at T1 outpatient group

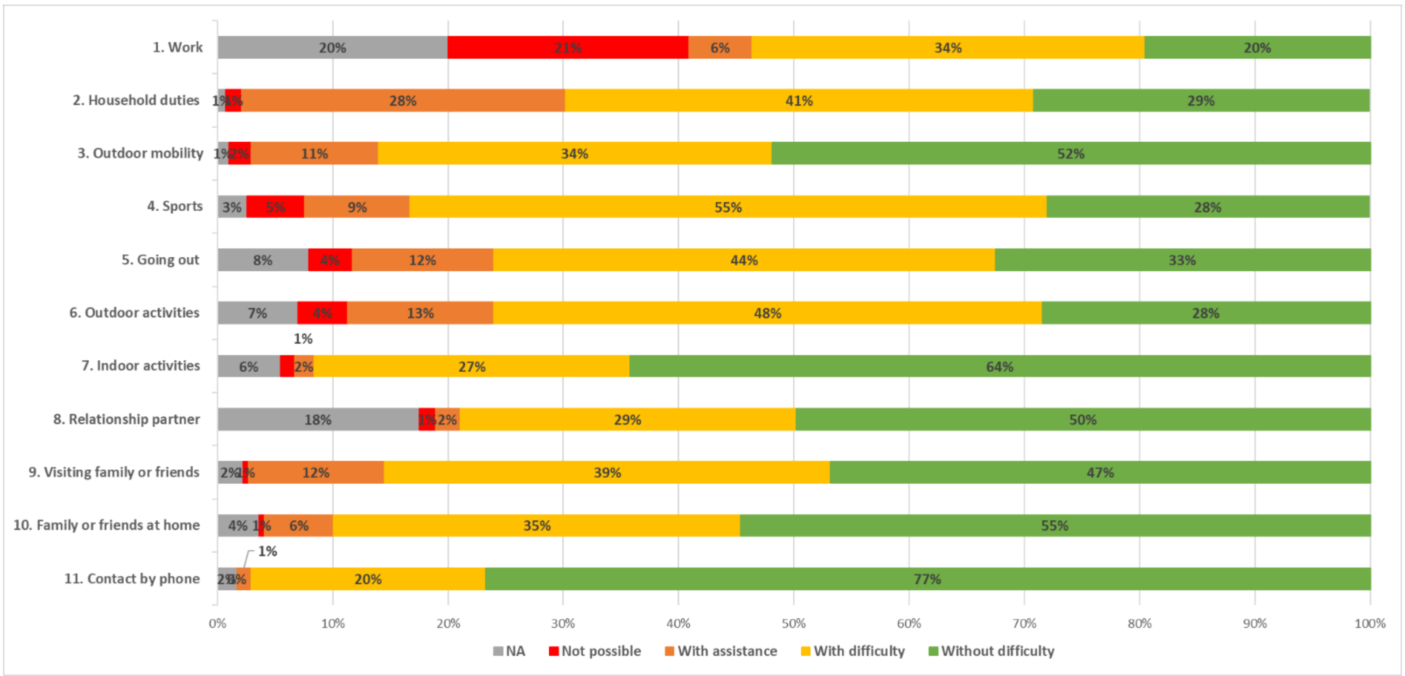

Fig 5. PROMIS-APS-SF at T0 inpatient group

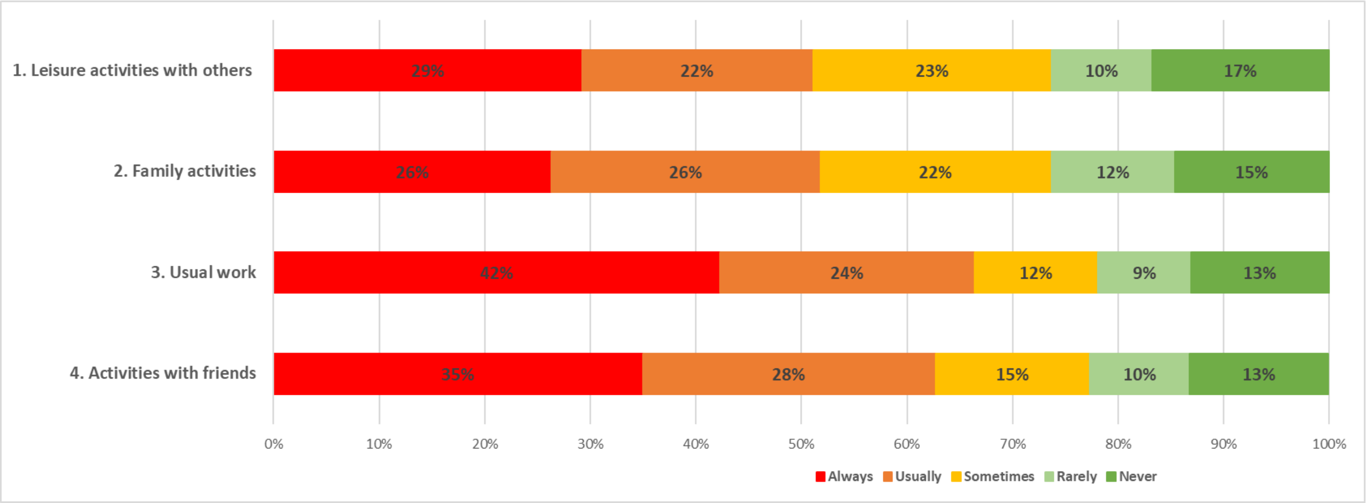

Fig 6. PROMIS-APS-SF at T1 inpatient group

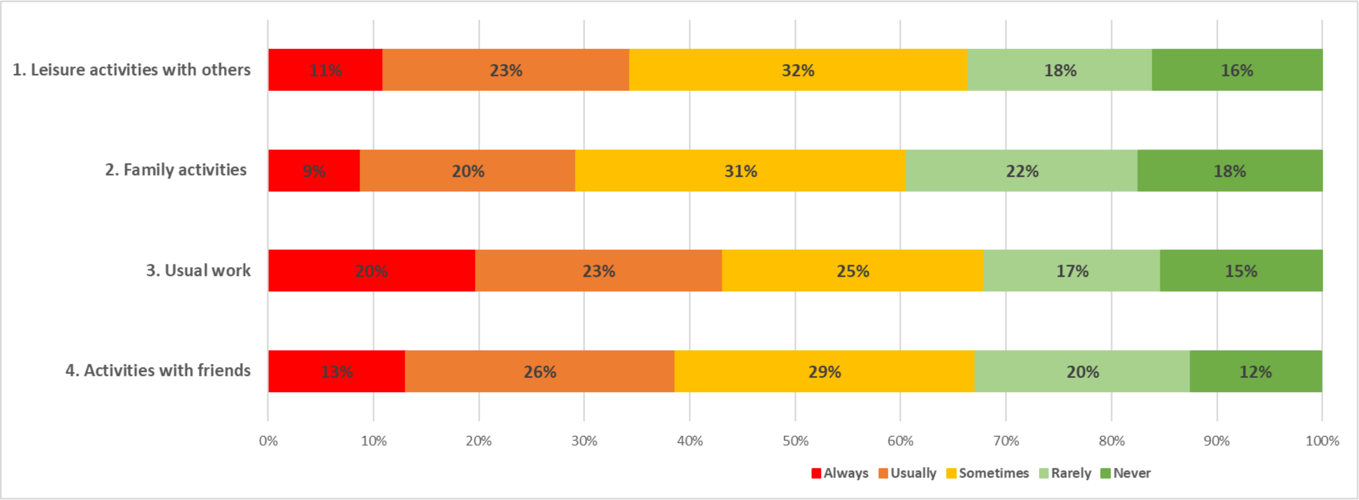

Fig 7. PROMIS-APS-SF at T0 outpatient group

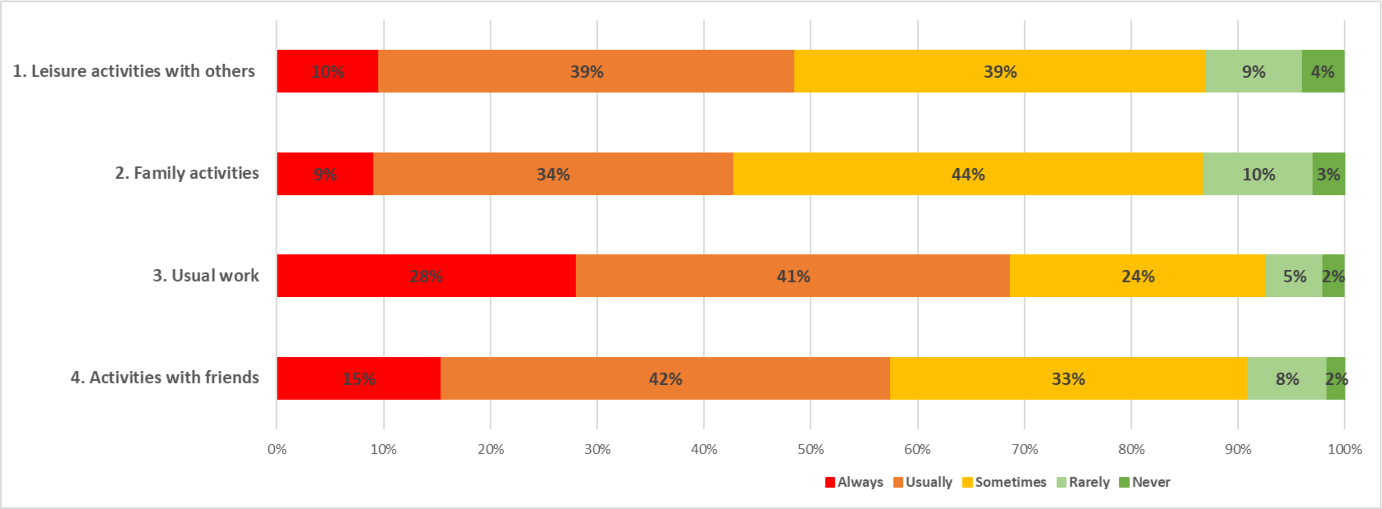

Fig 8. PROMIS-APS-SF at T1 outpatient group

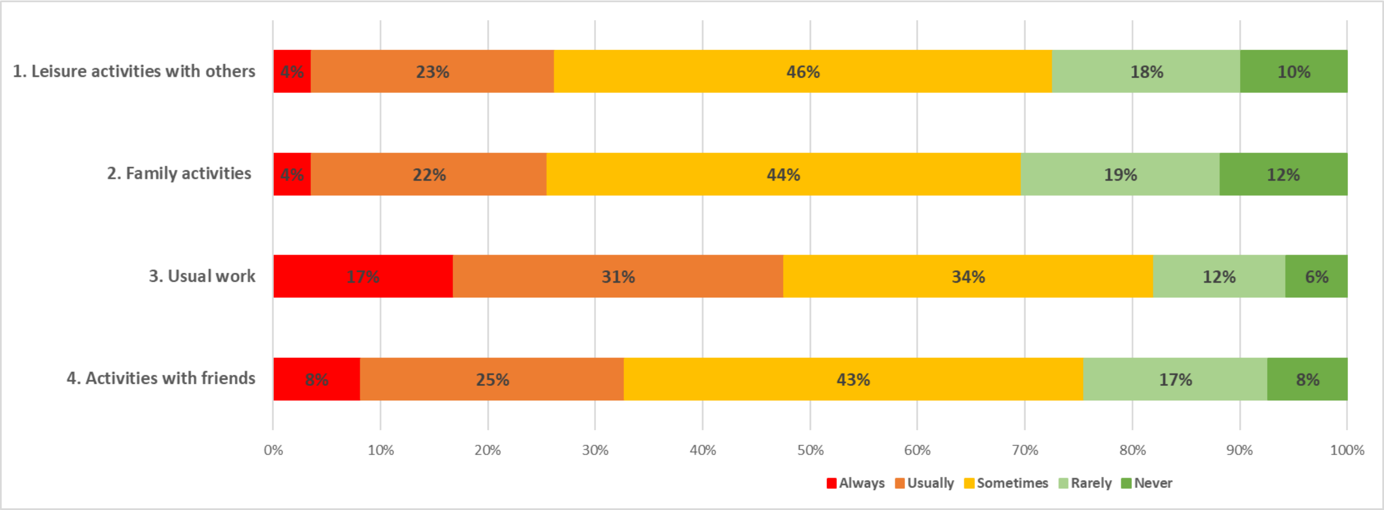

Article name:

Comparison of measurement properties between USER-Participation Restriction subscale and PROMIS® Ability to Participate in Social Roles and Activities in in- and outpatient rehabilitation populations

Journal name:

Quality of Life Research

Author names and affiliations:

B.M.P. Mourits, MSc[1]; E.W.M. Scholten, PhD[1,2]; J.A. de Graaf, (MD) PhD[1,2]; R.J.E.M. Smeets, (MD) PhD[3,4,5]; J. Nachtegaal, PhD[6]; C.E. de Boer, (MD)[7]; I.J.W. van Nes, (MD) PhD[8]; M. F. Reneman, (MD) PhD[9]; D.M. Oosterveer, (MD) PhD[10,11]; L. Valk-Kleibeuker, (MD)[12]; E.W.J. Agterhof, (MD)[13]; L.D. Roorda, (MD, PT) PhD[14]; J.M.A. Visser-Meily, (MD) PhD[1,2]; M.W.M. Post, PhD[1]

[1] Center of Excellence for Rehabilitation Medicine, UMC Utrecht Brain Center, University Medical Center Utrecht, and De Hoogstraat Rehabilitation, Utrecht, the Netherlands;
[2] Department of Rehabilitation, Physical Therapy Science & Sports, UMC Utrecht Brain Center, University Medical Center Utrecht, the Netherlands;
[3] Department of Rehabilitation Medicine, Care and Public Health Research Institute, Faculty of Health, Medicine & Life Sciences, Maastricht University, Maastricht, Netherlands;
[4] CIR Clinics In Revalidatie, Eindhoven, Netherlands;
[5] Pain in Motion International Research Group, Maastricht, Netherlands;
[6] Heliomare Rehabilitation Center, Research and Development, Wijk aan Zee, Netherlands
[7] Merem Medical Rehabilitation, Hilversum, The Netherlands;
[8] Department of Rehabilitation, Sint Maartenskliniek, Nijmegen, Netherlands;
[9] Department of Rehabilitation Medicine, Center for Rehabilitation, University Medical Center Groningen, University of Groningen, Groningen, Netherlands;
[10] Basalt, Leiden/The Hague, The Netherlands;
[11] Department of Rehabilitation Medicine, Alrijne Hospital, Leiden, The Netherlands;
[12] Department of Rehabilitation Medicine, Maasstad Hospital, Rotterdam, The Netherlands;
[13] De Hoogstraat Rehabilitation, Utrecht, the Netherlands;
[14] Amsterdam Rehabilitation Research Center, Reade, Amsterdam, The Netherlands.

Corresponding author:

E.W.M. Scholten

Center of Excellence for Rehabilitation Medicine, UMC Utrecht Brain Center, University Medical Center Utrecht, and De Hoogstraat Rehabilitation, Utrecht, the Netherlands

Address: UMC Utrecht Brain Center, Heidelberglaan 100, 3584 CX, Utrecht, The Netherlands

Email: E.Scholten-2@umcutrecht.nl
